# The Relationship of Th_1_/Th_2_ Cytokine Polarization at Parturition in Cows and SOCS3 Level With Some Postpartum Diseases

**DOI:** 10.1002/vms3.70137

**Published:** 2025-01-10

**Authors:** Oznur Yilmaz Koc, Ali Risvanli

**Affiliations:** ^1^ Department of Obstetrics and Gynecology Faculty of Veterinary Medicine Siirt University Siirt Turkey; ^2^ Department of Obstetrics and Gynecology Faculty of Veterinary Medicine Fırat University Elazığ Turkey; ^3^ Department of Obstetrics and Gynecology Faculty of Veterinary Medicine Kyrgyz‐Turkish Manas University Bishkek Kyrgyzstan

**Keywords:** cow, parturition, SOCS3, Th_1_/Th_2_ polarisation

## Abstract

Th_1_/Th_2_ polarisation and suppressor of cytokine signalling‐3 (SOCS3) are important indicators of the humoral and cellular immune system activity in cows. The aim of this study was to determine the correlation of postpartum diseases with the levels of Th_1_/Th_2_ polarisation and SOCS3 at the time of parturition. The study examined 180 cows (90 with normal parturition [NP] and 90 with dystocia [D]). Blood samples were taken from the cows once at the time of calving. Two subgroups were created among cows with NP: those without the postpartum disease (NP [−], *n* = 45) and those with postpartum disease (NP [+], *n* = 45). Likewise, two subgroups were created among D cows: those without postpartum disease (D [−], *n* = 45) and those with postpartum disease (D [+], *n* = 45). Cytokine analyses were performed using species‐specific commercial ELISA kits. In the NP (−) group, it was found that Th_1_/Th_2_ cytokine polarisation was in the Th_1_ direction due to the increase in the concentration of IFN‐γ, TNF‐α and IL‐2 in four subgroups grouping with different types of parturition and diseases. It was concluded that it would be appropriate to strengthen cellular immunity. In cases of postpartum diseases, Th_1_/Th_2_ polarisation shifted towards Th_2_ due to the increase in IL‐4 and IL‐5 concentrations in cows that performed NP and developed mastitis in the postpartum period. These results suggest that it would be beneficial to support the Th_2_ aspect (i.e. humoral immunity) in cows that have undergone NP and develop mastitis in the postpartum period.

## Graphical abstract

This study examined the relationship between Th_1_/Th_2_ polarisation and SOCS3 levels with postpartum diseases in cows. An increase in polarisation towards Th_1_ was observed in cows that gave parturition normally and did not have any disease, and an increase towards Th_2_ was observed in cows that had mastitis after giving parturition. The findings highlight the importance of immune support strategies.

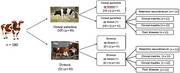

## 1 Introduction

The periparturient period includes the 3 weeks before and 3 weeks after parturition and involves a very complex process with metabolic and hormonal changes, inflammation and immunity in dairy cows (Islam et al. 2013; Pascottini, Leroy, and Opsomer 2020; Safak, Yilmaz, and Risvanli 2023a). Health in the periparturient period is an important indicator of fertility performance in subsequent periods (Ferguson 2005). Many researchers have different definitions of this period, which is characterised by sudden physiological changes (Grummer 1995; Drackley 1999; Trevisi et al. 2011). However, they have minimised many factors and promoted a few critical points. These include issues such as negative energy balance (NEB), oxidative stress, inflammation and weakening of the immune system (Trevisi and Minuti 2018).

Although most of the changes in these issues are expressed by focusing on the postpartum period, changes in the immune system are more important in the prepartum period (Trevisi and Minuti 2018). The differences in the hormonal, metabolic, immune or neurological systems during the periparturient period increase the risk of metabolic disorders and of infectious diseases such as retention secundinarum, metritis, and mastitis (Ingvartsen and Moyes 2015). These diseases occur in the periparturient period and adversely affect the reproductive performance of the animal during lactation, causing major economic losses (Roche 2006; Gençoğlu 2012; Risvanli et al. 2021; Safak, Yilmaz, et al. 2022).

Dairy cows are generally protected against disease by a very strong immune system. Physiological and metabolic changes associated with the onset of parturition and lactation in the periparturient period are associated with dysregulation of the appropriate immune and inflammatory response. NEB is a result of reduced dry matter intake and insufficient nutrient intake during the transition period and has serious negative effects on the animals' defence mechanism in the early stages of lactation (Sordillo 2016; Yilmaz and Risvanli 2020).

Cytokines have an important place in postpartum immunology in cows. Cytokines can be used to detect problems that may occur during the postpartum period and to determine the immune status of the animal. Helper T (Th)_1_ cells stimulate the phagocytic‐mediated destruction and killing of microorganisms, thus constituting the most important building block of cellular immunity. The most important cytokine produced by Th_1_ cells is interferon gamma (IFN‐γ). The function of Th_1_ cells is to activate macrophages, which are involved in the effective destruction of ingested microorganisms (Abbas, Lichtman, and Pillai 2015; Kunkl et al. 2020).

Helper T_2_ cells are responsible for the production of interleukin 4 (IL‐4), which initiates the production of immunoglobulin (IgE) antibodies and IL‐5, which stimulates the production of eosinophils. At the same time, Th_2_ cell cytokines activate macrophages. Th_1_‐mediated activation increases the ability of macrophages to eliminate microorganisms, whereas Th_2_‐mediated macrophage activation increases functions such as the production of extracellular matrix proteins that are effective in tissue repair (Abbas, Lichtman, and Pillai 2015; Wynn 2004; Walker and McKenzie 2017). Some of the cytokines produced by Th_2_ cells, including interleukin‐4, IL‐10, and IL‐13, have an effect of suppressing the microbicidal activity of macrophages and Th_1_ cell‐mediated immunity. Therefore, the effect of cell‐mediated immune responses against a microorganism can be achieved by maintaining a balance between the activation of Th_1_ and Th_2_ cell responses against the microorganism (Abbas, Lichtman, and Pillai 2015).

This balance determines the outcome of many infections. Th_1_ cells increase the defence against intracellular microorganisms, while Th_2_ cells suppress it. It is generally accepted that Th_1_ cytokines are responsible for cellular immunity, while Th_2_ cytokines are responsible for humoral immunity. The ratio of Th_1_ and Th_2_ determines the progression of an infectious agent. Although Th_1_ cells are responsible for the production of cytokines such as IFN‐γ, IL‐2 and tumour necrosis factor alpha (TNF‐α), Th_2_ cells are responsible for the production of cytokines such as IL‐4, IL‐5 and IL‐10 (Zhu and Paul 2008; Abbas, Lichtman, and Pillai 2015; Yang et al. 2019).

Th_1_/Th_2_ cytokine polarisation and suppressor of cytokine signalling‐3 (SOCS3) are important indicators of humoral and cellular immune system activity. The aim of the present study was to determine the relationship of postpartum diseases with Th_1_/Th_2_ cytokine polarisation and SOCS3 levels in cows.

## 2 Materials and Methods

### 2.1 Establishment of Study Groups

Ethical approval for this study was obtained from Siirt University Animal Experiments Local Ethics Committee (dated 23 September 2020, protocol number 2020/12, decision number 2020/03‐06). The study included cows that started giving parturition in commercial dairies in Siirt Province, Türkiye. The clinically healthy cows (*n* = 180) had different ages and breeds, weighed 450–500 kg and had body condition scores (BCS) of 3.0–3.5. The required minimum sample number was determined as 180 using an effect size of 0.25, alpha of 0.05 and power of 0.80 (Cohen 1988).

One blood sample was collected in the second stage of parturition to ensure uniformity from 500 cows of different ages and breeds that had completed their pregnancy period. These cows were then divided into two groups: those with normal parturition (NP) and those with dystocia (D). Each group was divided into two subgroups: those with postpartum disease (+) and those without it (−). One hundred eighty cows were included with 45 cows in each group.

**FIGURE 1:**
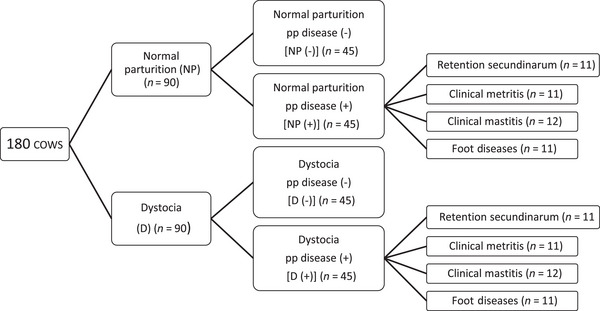
Grouping of study material.

The animals were then observed for a 15‐day postpartum period. Cows that developed retention secundinarum, clinical metritis, foot disease and clinical mastitis during this period were included in the group of animals that developed postpartum disease. If more than one disease occurred in the same cow during that 15‐day postpartum period, it was excluded from the study. The grouping of the cows was done as shown in Figure 1.

Grouping was performed according to the type of parturition. At the end of the normal pregnancy period specific to the species, parturitions that occurred without any negative effects on the mother and calf and without the need for assistance were called NPs. Dystocia was defined as cases in which NP could not take place within the time period specific to the species or did not occur without any assistance and put the lives of the mother or the calf in danger (Musal and Köker 2019).

The following criteria were taken into consideration in the grouping of cows with postpartum problems according to diseases. Retention secundinarum was defined based on the criteria described by Gencoglu (2012), as cases in which the offspring's membranes could not be expelled within the first 24 h. Metritis was diagnosed based on the criteria described by Sheldon et al. (2006) in animals that showed purulent or mucopurulent discharge in the vagina within the first 15 days of parturition, had fluid detected in the uterus by rectal or ultrasonographic examination, and did not show any general condition disorders. Mastitis was diagnosed based on the criteria described by Ruegg and Erskine (2014), in animals that have milk defects in at least one udder within the first 15 days of parturition as well as have symptoms such as redness, pain, temperature increase and hyperaemia in an udder.

### 2.2 Cytokine and SOCS3 Analysis

Blood samples were taken from all cows at the time of parturition in 10 mL non‐anticoagulant tubes. After clotting, serum samples were obtained by centrifuging at 3000 rpm for 10 min and stored in 2 mL eppendorf tubes at a temperature of −80°C until analysis. Commercial species‐specific bovine ELISA kits were used to determine the levels of Th_1_ (IFN‐γ, IL‐2, TNF‐α) and Th_2_ (IL‐4, IL‐5, IL‐10) cytokines and SOCS‐3 in the collected blood serum. The catalogue numbers of the commercial kits used are as follows: Bovine IFN‐γ (catalogue no: SEA049Bo, all USCN Life Science Inc., Wuhan, China), Bovine IL‐2 (catalogue no: SEA073Bo, all USCN Life Science Inc.), Bovine TNF‐α (catalogue no: SEA133Bo, all USCN Life Science Inc.), Bovine IL‐4 (catalogue no: SEA077Bo, all USCN Life Science Inc.), Bovine IL‐5 (catalogue no: SEA078Bo, all USCN Life Science Inc.), Bovine IL‐10 (catalogue no: SEA056Bo, all USCN Life Science Inc.) and Bovine SOCS‐3 (catalogue no: SEB684Bo, all USCN Life Science Inc.). According to the manufacturer, the minimum detectable concentration of bovine IFN‐ γ, IL‐2, TNF‐α, IL‐10, IL‐5, IL‐4, IL‐5, SOCS3 are less than 5.8, 5.9, 3.1, 5.6, 5.7, 6.1 and 6.4 pg/mL, respectively. The inter‐ and intra‐assay coefficients of variation (CV) for all examined cytokines were < 12% and < 10%, respectively (Brodzki et al. 2015). The application stages of the ELISA test were carried out according to the manufacturer's instructions and methods in the literature. After all stages were completed, the results were read by an automatic microtiter plate reader (Bio Tek Instruments, USA) at 450 nm (Safak and Risvanli 2021).

### 2.3 Statistical Analysis

The programme SPSS 22.0 (Statistical Package for the Social Sciences for Windows SPSS 22.0 Edition for Windows, Chicago, IL, USA) was used for statistical analysis of the data. The normality of the data distribution was examined visually (histogram and probability graphs) and with analytical methods (Kolmogorov–Smirnov/Shapiro–Wilk). It was first assessed whether the data followed a normal distribution. Since the data did not meet parametric assumptions in the multi‐group comparison, the Kruskal–Wallis test, a non‐parametric test, was applied. A normality test was first conducted for the binary groups. For data that met parametric assumptions, an independent *t*‐test was applied, while the Mann–Whitney *U* test was used for data that did not meet these assumptions.

## 3 Results

Table 1 shows the levels of serum cytokine between the disease (+) and disease (−) groups according to the parturition. The highest serum level of IFN‐γ was detected in the NP (−) group (506.64 ± 97.54 pg/mL). There was no statistically significant difference between the disease (−) group and the D (−) group that had parturition normally, but a significant difference was observed between the NP (−) group and the NP (+) and D (+) groups (*p* < 0.001). The TNF‐α level was normal in the NP (−) (574.29 ± 34.39 pg/mL), NP (+) (202.96 ± 38.66 pg/mL), D (−) (79.38 ± 14.52 pg/mL) and D (+) (55.01 ± 13.52 pg/mL) groups (*p* < 0.001), but there was no statistical difference between the D (−) and D (+) groups (*p* > 0.05).

**TABLE 1:** Serum cytokine levels between disease (−) and disease (+) groups according to type of parturition.

Cytokine	NP (−) (*n* = 45)		NP (+) (*n* = 45)		D (−) (*n* = 45)		D (+) (*n* = 45)		*p*
	𝑥̅ ± 𝑠	Median	𝑥̅ ± 𝑠	Median	𝑥̅ ± 𝑠	Median	𝑥̅ ± 𝑠	Median	
IFN‐γ (pg/mL)	506.64 ± 97.54^a^	248	167.91 ± 50.6^b^	58.19	226.40 ± 58.6^ab^	68.60	122.26 ± 28.18^b^	65.35	< 0.001
TNF‐α (pg/mL)	574.29 ± 34.39^a^	671.22	202.96 ± 38.66^b^	102.70	79.38 ± 14.52^c^	23.46	55.01 ± 13.52^c^	17.34	< 0.001
IL‐2 (pg/mL)	1591.30 ± 241^a^	1097	629.89 ± 163.9^b^	335.90	620.82 ± 102.8^b^	280.30	418.27 ± 59.88^b^	304.40	< 0.001
IL‐4 (pg/mL)	1386.99 ± 53.4^a^	1332	887.37 ± 77.59^b^	835.30	928.37 ± 79.14^b^	909.20	903.32 ± 73.05^b^	947	< 0.001
IL‐5 (pg/mL)	192.50 ± 17.44	196.95	162.92 ± 17.01	131.80	261.16 ± 188	43.50	41.50 ± 6.69	25.32	> 0.05
IL‐10 (pg/mL)	787.74 ± 83.35^a^	797.80	239.65 ± 62.02^bc^	57.03	244.73 ± 25.38^c^	194.7	325.67 ± 28.44^b^	367.35	< 0.001
SOCS3 (ng/mL)	12.98 ± 2.69^a^	4.82	3.22 ± 1.29^b^	1.08	7.14 ± 2.19^ab^	1.42	2.54 ± 0.98^b^	1.16	< 0.001

*Note*: ^a, b, c^: The difference between groups with different letters on the same line is statistically significant at *p* > 0.05.

Abbreviations: D, dystocia; IFN‐γ, interferon gamma; IL, interleukin; NP, normal parturition; TNF‐α, tumour necrosis factor‐alpha.

The results of the Th_1_/Th_2_ polarisation between the groups are given in Table 2. The levels of IFN‐γ, TNF‐α and IL‐2 were found to be higher in the NP (−) group compared to the other groups.

**TABLE 2:** Th_1_/Th_2_ cytokine polarisation between groups.

	Th_1_			Th_2_		
	IFN‐γ	TNF‐α	IL‐2	IL‐4	IL‐5	IL‐10
NP (−) (*n* = 45)	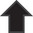	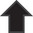	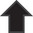	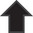		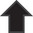
NP (+) (*n* = 45)				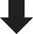		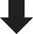
D (−) (*n* = 45)					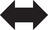	
D (+) (*n* = 45)	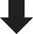	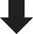	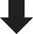		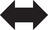	

*Note*: 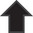: high concentration; 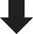: low concentration; 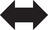: concentration does not change.

Table 3 shows the serum cytokine levels of animals with metritis according to the type of parturition. A statistically significant difference was observed in the serum level of IL‐5 in the NP group (167.34 ± 21.69 pg/mL) compared to the D group (66.09 ± 21.95 pg/mL; *p* < 0.01).

**TABLE 3:** Serum cytokine levels in animals with metritis according to the type of parturition.

Cytokine	NP (*n* = 11)		D (*n* = 11)		*p*
	𝑥̅ ± 𝑠𝑥̅	Median	𝑥̅ ± 𝑠𝑥̅	Median	
IFN‐γ (pg/mL)	359.97 ± 168.45	58.19	190.09 ± 104.91	60.70	> 0.05
TNF‐α (pg/mL)	238.09 ± 52.55	243.43	109.37 ± 44.47	18.71	> 0.05
IL‐2 (pg/mL)	911.47 ± 396.75	465.30	440.01 ± 117.81	346.20	> 0.05
IL‐4 (pg/mL)	946.60 ± 130.18	834	900.93 ± 106.32	947	> 0.05
IL‐5 (pg/mL)	167.34 ± 21.69	150	66.09 ± 21.95	34.42	< 0.001
IL‐10 (pg/mL)	487.58 ± 188.68	174.40	433 ± 46.20	381.95	> 0.05
SOCS3 (ng/mL)	6.21 ± 4.50	1.28	4.94 ± 3.69	1.46	> 0.05

Abbreviations: D, dystocia; IFN‐γ, interferon gamma; IL, interleukin; NP, normal parturition; TNF‐α, tumour necrosis factor‐alpha.

Table 4 shows the serum cytokine levels of animals with clinical mastitis according to the type of parturition. Serum TNF‐α and IL‐4 levels were found to be significantly higher in the NP group (169.73 ± 72.49 pg/mL; 870.57 ± 134.46 pg/mL) than the D group (29.45 ± 19.15 pg/mL; 555.76 ± 179.23 pg/mL; *p* < 0.05). The serum IL‐5 and IL‐10 levels were found to be higher in the NP group (157.8 ± 29.25 and 385.9 ± 64.67 pg/mL, respectively) than the D group (*p* < 0.01). No significant difference was detected in serum IFN‐γ, IL‐2 and SOCS‐3 levels (*p* > 0.05).

**TABLE 4:** Serum cytokine levels of animals with clinical mastitis according to parturition type.

Cytokine	NP (*n* = 11)		D (*n* = 11)		*p*
	𝑥̅ ± 𝑠𝑥̅	Median	𝑥̅ ± 𝑠𝑥̅	Median	
IFN‐γ (pg/mL)	84.13 ± 18.93	71.5	80.93 ± 14.11	67	> 0.05
TNF‐α (pg/mL)	169.73 ± 72.49	19.57	29.45 ± 19.15	10.70	< 0.05
IL‐2 (pg/mL)	397.11 ± 84.07	386.4	417.83 ± 126.41	300.8	> 0.05
IL‐4 (pg/mL)	870.57 ± 134.46	748.6	555.76 ± 179.23	472.40	< 0.05
IL‐5 (pg/mL)	157.8 ± 29.25	124	29.76 ± 5.69	27.2	< 0.001
IL‐10 (pg/mL)	129.29 ± 57.48	78	385.9 ± 64.67	396.5	< 0.001
SOCS3 (ng/mL)	1.44 ± 0.35	1.08	1.02 ± 0.18	0.75	> 0.05

Abbreviations: D, dystocia; IFN‐γ, interferon gamma; IL, interleukin; NP, normal parturition; TNF‐α, tumour necrosis factor‐alpha.

Table 5 shows the Th_1_/Th_2_ cytokine polarisation in cows with clinical mastitis according to the type of parturition. An increase in the concentration of IL‐4 and IL‐5 was observed in NP (+) group. Accordingly, it was determined that the Th_1_/Th_2_ cytokine polarisation balance in the NP (+) group was in the Th_2_ direction.

**TABLE 5:** Th_1_/Th_2_ cytokine polarisation in those with clinical mastitis according to the type of parturition.

	Th_1_			Th_2_		
	IFN‐γ	TNF‐α	IL‐2	IL‐4	IL‐5	IL‐10
NP(+) (*n* = 12)	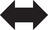	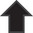		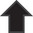	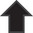	
D(+) (*n* = 12)			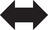			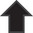

*Note*: 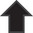: high concentrations; 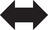: concentration does not change.

Table 6 shows the serum cytokine levels of animals with retention secundinarum according to the type of parturition. The serum IL‐5 level was found to be higher in the NP group (127.67 ± 30.27 pg/mL) than the D group (26.45 ± 3.87 pg/mL; *p* < 0.05). There was no significant difference between the groups in serum IFN‐γ, TNF‐α, IL‐2, IL‐4, IL‐10 and SOCS‐3 levels (*p* > 0.05). When we looked at the Th_1_/Th_2_ cytokine polarisation in cows that formed retention secundinarum according to the type of parturition, it was determined that the polarisation balance did not change, and only the IL‐5 concentration increased in NP (+) group.

**TABLE 6:** Serum cytokine levels of animals formed with RS according to the type of parturition.

Cytokine	NP (*n* = 11)		D (*n* = 11)		*p*
	𝑥̅ ± 𝑠𝑥̅	Median	𝑥̅ ± 𝑠𝑥̅	Median	
IFN‐γ (pg/mL)	85.31 ± 18.36	52.79	102.33 ± 37.32	65.35	> 0.05
TNF‐α (pg/mL)	247.52 ± 75.53	137.2	48.46 ± 11.84	47.79	> 0.05
IL‐2 (pg/mL)	331.05 ± 105.43	222	487.67 ± 119.14	304.4	> 0.05
IL‐4 (pg/mL)	741.10 ± 201.11	343.7	1115.59 ± 138.54	1024	> 0.05
IL‐5 (pg/mL)	127.67 ± 30.27	115.60	26.45 ± 3.87	25.06	< 0.05
IL‐10 (pg/mL)	183.53 ± 68.31	16.36	220.59 ± 44.57	170.6	> 0.05
SOCS3 (ng/mL)	1.35 ± 0.54	0.57	1.95 ± 0.58	1.59	> 0.05

Abbreviations: D, dystocia; IFN‐γ, interferon gamma; IL, interleukin; NP, normal parturition; TNF‐α, tumour necrosis factor‐alpha.

Table 7 shows the serum cytokine levels of the animals with foot disease according to the type of parturition. Serum IL‐5 levels were higher in NP groups (176.8 ± 47.78 pg/mL; *p* < 0.05), whereas IL‐10 levels were higher in D animals (324.18 ± 35.38 pg/mL; *p* < 0.001). No significant difference was found in serum IFN‐γ, TNF‐α, IL‐2, IL‐4 and SOCS‐3 levels (*p* > 0.05).

**TABLE 7:** Serum cytokine levels of animals with foot diseases according to the type of parturition.

Cytokine	NP (*n* = 11)		D (*n* = 11)		*p*
	𝑥̅ ± 𝑠𝑥̅	Median	𝑥̅ ± 𝑠𝑥̅	Median	
IFN‐γ (pg/mL)	44.85 ± 6.30	35.41	97.95 ± 24.96	60.99	> 0.05
TNF‐α (pg/mL)	61.15 ± 27.24	16.02	16.50 ± 1.50	16.93	> 0.05
IL‐2 (pg/mL)	272.9 ± 87.1	113.7	215.7 ± 45.79	173.25	> 0.05
IL‐4 (pg/mL)	898.63 ± 144.72	873	943.75 ± 79.24	1024	> 0.05
IL‐5 (pg/mL)	176.8 ± 47.78	124	34.10 ± 8.55	24.73	< 0.05
IL‐10 (pg/mL)	45.08 ± 11.47	32.72	324.18 ± 35.38	367.65	< 0.001
SOCS3 (ng/mL)	1.285 ± 0.37	0.92	0.81 ± 0.12	0.68	> 0.05

Abbreviations: D, dystocia; IFN‐γ, interferon gamma; IL, interleukin; NP, normal parturition; TNF‐α, tumour necrosis factor‐alpha.

## 4 Discussion

Cytokines are soluble proteins that are involved in a series of biological processes, including inflammation and immunity. They not only play an important role in controlling and regulating an organism's reactions to foreign antigens and agents but also take part in the local and systemic inflammatory response by regulating intercellular relations (Baykal, Karaayvaz, and Kutlu 1998; Bannerman 2009; Risvanli et al. 2019). IFN‐γ plays a role in the communication between innate and adaptive immunity and is also critical in the development of host immunity against intracellular pathogens (Bannerman 2009; Safak, Yilmaz, and Risvanli 2023b).

Vitenberga‐Verza et al. (2022) investigated cytokine levels in cows with clinical and subclinical mastitis for 6 days, and IFN‐γ levels were found to be high in cows with clinical mastitis. However, they reported that IFN‐γ decreases with the elimination of symptoms. A study on foot diseases found that bacterial infection leads to an increase in IFN‐γ levels (Nazifi et al. 2012). The study found no significant difference in blood IFN‐γ levels at parturition between NP and D groups. However, D group with foot disease had higher IFN‐γ concentrations.

It is reported that plasma TNF‐α increases dramatically on the day of parturition in cows, thereby playing an important role in the excretion of foetal membranes (Boro et al. 2015). Karan Fries and Sahiwal cows had plasma samples taken on Days 21, 14 and 7 prepartum, calving day, and 1 and 2 days postpartum. Plasma TNF‐α levels increased towards calving and were higher on the day of calving. In the same study, significantly lower TNF‐α concentrations were observed on the day of calving and the day before calving in cows that developed retention secundinarum compared to cows that did not in both breeds (Boro et al. 2015). In the study conducted by Chandra et al. (2012), low TNF‐α concentrations were observed, a finding that is consistent with the results reported by Boro et al. (2015).

In our study, no statistically significant difference could be detected in the TNF‐α level between NP and D groups that developed retention secundinarum. However, the TNF‐α level was found to be higher in NP compared to D animals. Studies have reported that TNF‐α plays an important role in the removal of foetal membranes, which is consistent to the increase in the NP (−) group in our study. Galvão (2013) reported that serum TNF‐α concentration was lower in cows with metritis compared to healthy cows at calving and Days 7 and 21 postpartum. Cui et al. (2019) reported that metritis that develops during the postpartum period may be linked to TNF‐α concentrations on Day 7 prepartum, calving day, and Day 7 postpartum. In this study, the TNF‐α concentration was reported to be higher on the seventh day prepartum compared to the postpartum days. In contrast to these studies, Avcılar et al. (2023) reported that TNF‐α, IL‐1β, IL‐6 and IL‐10 concentrations increased in animals with acute septic metritis in the fourth week before parturition.

In response to tissue damage and microbial infection, immune cells invade the uterus and mammary gland during involution or parturition and produce high levels of pro‐inflammatory cytokines such as IL‐1β, IL‐6 and TNF‐α (Kuhla 2020). In our study, the TNF‐α concentration was found to be higher in groups where disease did not occur. Safak and Risvanli (2022) found the TNF‐α concentration to be lower in milk from cows with subclinical mastitis with high somatic cell count (SCC). Nazifi et al. (2012) reported that the concentrations of inflammatory mediators TNF‐α and IFN‐γ increased in response to bacterial infection.

It has been suggested that there is an increase in TNF‐α concentration due to inflammation and endotoxemia in cows with foot disease during the prepartum period (Zhang et al. 2015). TNF‐α is known to increase with the severity of inflammation. In this study, it was determined that TNF‐α levels were not different according to the type of parturition for the animals with foot disease. There have been differences between studies, which may be due to individual characteristics of the cows, their nutritional status and seasonal changes.

Shaheen et al. (2020) investigated IL‐10 levels in cows with clinical mastitis or subclinical mastitis and healthy dairy cows and found that this cytokine was significantly lower in animals with clinical mastitis compared to the other two groups. Bochniarz et al. (2017) found that the serum level of anti‐inflammatory IL‐10 in cows with subclinical mastitis was significantly lower than in healthy cows. Safak, Risvanli, and Asci‐Toraman (2022) examined the relationship between SCC and IL‐10 levels and reported that there was no significant difference between SCC and IL‐10.

In contrast to these studies, Shen et al. (2020) compared the healthy cows with cows with subclinical and clinical mastitis and found that the IL‐10 level was significant in cows with subclinical mastitis, and it was significantly higher in cows with clinical mastitis. They reported that the reason for this may be that inflammatory symptoms are more pronounced in cows with clinical mastitis, and the increase in acute pro‐inflammatory factors may damage the udder tissues and even the whole body. In this study, when the serum cytokine levels of the animals with mastitis were analysed according to the type of parturition, the IL‐10 level was found to be higher in the D group. This is thought to be due to greater stress in the D group compared to the NP group.

Interleukin‐10 plays an important role in stimulating Th_1_ polarisation and changes the Th_1_/Th_2_ balance towards Th_2_ by suppressing IFN‐γ and IL‐12 (Mocellin et al. 2004). Islam et al. (2013) examined IL‐10 levels in blood serum collected on Day 15 prepartum, calving day, and Days 15 and 30 postpartum. Serum IL‐10 levels of cows that developed clinical metritis in the postpartum period were found to be higher than those of healthy cows on Day 15 prepartum, calving day, and Day 15 postpartum. Another study reported an increase in IL‐10 concentration on the day of calving in blood serum samples taken on the day of calving and Days 14 and 21 postpartum when animals with clinical metritis and healthy animals were compared (Galvão 2013).

In our study, IL‐10 levels were found to be the highest in the NP (−) group in the serum taken at the time of parturition, and there was no difference between IL‐10 levels between modes of parturition. It is known that the levels of cytokines are affected by many factors, such as air temperature, breed, age, rations and stress. The reason why there was no significant difference in this study is thought to be the different age and breeds of the animals from those in other studies, as well as feeding with different rations.

IL‐4 is an important cytokine of Th_2_ cells that mediates adaptive humoral immunity and functions as both an inducer and effector of cells. Furthermore, IL‐4 antagonises the macrophage‐activating effects of IFN‐γ and thus inhibits cell‐mediated immune reactions. Bochniarz et al. (2017) suggested that IL‐4 levels were significantly increased in cows with subclinical mastitis compared to healthy cows. Safak, Risvanli, and Asci‐Toraman (2022) found that the IL‐4 level was lower in cows with mastitis caused by *Escherichia coli* and *Staphylococcus aureus* compared to healthy animals. In contrast to these studies, Fonseca et al. (2009) found no difference in IL‐4 concentrations in mastitic and healthy animals. In our study, the IL‐4 level was found to be higher in animals with disease, which parallels the study by Bochniarz et al. (2017).

Interleukin‐2 is a cytokine secreted by activated T lymphocytes that leads to replication and clonal differentiation of other helper and cytotoxic T cell populations, as well as the initiation of immune response and immunological memory (Zecconi et al. 2009). It was reported that the IL‐2 level was significantly lower in cows with retention secundinarum compared with healthy cows (Li et al. 2021). Šerstņova et al. (2022) reported that when mastitis and healthy cows were compared, milk IL‐2 concentrations were higher in mastitis cows and remained stable in measurements made for 3 days. However, another study indicated that in bovine mammary glands experimentally infected with *S. aureus*, IL‐2 concentration showed a significant decrease during the early stages of inflammation (Alluwaimi et al. 2003).

Cytokine signal suppressor receptor‐3 is a protein that negatively regulates the cytokine signalling pathway (Tamiya et al. 2011) and plays a fundamental role in maintaining the balance between the harmful and beneficial effects of cytokines (Linossi, Calleja, and Nicholson 2018). It inhibits Th_1_ differentiation and stimulates Th_2_ differentiation by suppressing the IL‐12/signal transducer and activator of transcription (STAT) 4 pathway. Furthermore, it inhibits STAT3 and prevents differentiation to Th_17_, which causes high pro‐inflammatory responses; therefore, inflammation begins in the absence of SOCS‐3 protein (Takahashi et al. 2011).

Cytokine signal suppressor receptor‐3 can be modulated by various inflammatory stimuli and inhibit the signalling interactions of immune molecules (Latvala et al. 2011). In our study, the SOCS‐3 level was higher in the disease‐free groups, which is consistent with this information. At the same time, the serum SOCS‐3 concentration was generally lower in animals with postpartum disease in the D group. It is thought that this situation may develop due to the suppression of the immune system due to the greater stress of D group than NP group and the higher release of cortisol.

## 5 Conclusion

In this study, it was found that Th_1_/Th_2_ cytokine polarisation was in the Th_1_ direction due to the increase in the concentrations of IFN‐γ, TNF‐α and IL‐2 in the NP (−) group. Therefore, it was concluded that it would be appropriate to support cellular immunity in such cases. To support cellular immunity, copper, zinc, vitamin A, selenium, vitamin E and vitamin D supplements in the form of D3 and D2 are recommended. Apart from these, it is recommended to organise feeding programmes to reduce blood ketone levels by preventing cows from entering NEB.

Th_1_/Th_2_ polarisation shifted towards Th_2_ due to the increase in IL‐4 and IL‐5 concentrations in NP cows that developed mastitis in the postpartum period. These findings suggest that it would be beneficial to support the Th_2_ aspect (i.e. humoral immunity) in cows with mastitis in NP and postpartum periods. It is thought that it would be beneficial to administer vaccines that keep Ig levels high, as well as *Corynebacterium cutis* lysate applications that cause increases in Ig levels, homeopathic medicines and various peptides to strengthen humoral immunity. Although it is not possible to determine a relationship between the serum level of SOCS‐3 at parturition and the type of parturition or postpartum‐shaped diseases, it is thought that further studies on SOCS‐3 will contribute to studies on the immune system in cows.

## Author Contributions

**Oznur Yilmaz Koc**: data curation, investigation, methodology, supervision, validation, visualisation, writing–original draft. **Ali Risvanli**: data curation, investigation, methodology, validation, writing–review and editing. All authors have read and approved the version of the article submitted for publication.

## Ethics Statement

Ethical approval for this study was obtained from Siirt University Animal Experiments Local Ethics Committee (dated 23 September 2020, protocol number 2020/12, decision number 2020/03‐06).

## Conflicts of Interest

The authors declare no conflicts of interest.

### Peer Review

The peer review history for this article is available at https://publons.com/publon/10.1002/vms3.70137.

## Data Availability

The authors have nothing to report.
